# A Core Response to the CDX2 Homeoprotein During Development and in Pathologies

**DOI:** 10.3389/fgene.2021.744165

**Published:** 2021-10-25

**Authors:** Victor Gourain, Isabelle Duluc, Claire Domon-Dell, Jean-Noël Freund

**Affiliations:** ^1^ Karlsruhe Institute of Technology, Institute of Biological and Chemical Systems, Karlsruhe, Germany; ^2^ Université de Strasbourg, Inserm, IRFAC / UMR-S1113, FHU ARRIMAGE, FMTS, Strasbourg, France

**Keywords:** homeobox gene, embryo, cancer, gene expression, chromatin targets

## Abstract

Whether a gene involved in distinct tissue or cell functions exerts a core of common molecular activities is a relevant topic in evolutionary, developmental, and pathological perspectives. Here, we addressed this question by focusing on the transcription factor and regulator of chromatin accessibility encoded by the Cdx2 homeobox gene that plays important functions during embryonic development and in adult diseases. By integrating RNAseq data in mouse embryogenesis, we unveiled a core set of common genes whose expression is responsive to the CDX2 homeoprotein during trophectoderm formation, posterior body elongation and intestinal specification. ChIPseq data analysis also identified a set of common chromosomal regions targeted by CDX2 at these three developmental steps. The transcriptional core set of genes was then validated with transgenic mouse models of loss or gain of function of Cdx2. Finally, based on human cancer data, we highlight the relevance of these results by displaying a significant number of human orthologous genes to the core set of mouse CDX2-responsive genes exhibiting an altered expression along with CDX2 in human malignancies.

## Introduction

That evolution makes new out of old suggests the existence of shared properties between the functions of a given gene at its different times or sites of action. The homeobox gene encoding the CDX2 transcription factor allows addressing this assumption since it drives three major developmental processes in mammals. At the blastula stage, Cdx2 is pivotal during the segregation of pluripotent cells into the first two lineages by acting downstream of the lineage allocation process between trophectodermal and inner mass cells to repress Oct4 and Nanog in the trophectoderm (Niwa et al., 2005; Strumpf et al., 2005; Ralston and Rossant, 2008). Then, Cdx2 actively participates in axial posterior body growth at gastrulation through a convergent effect with T-Brachyury to maintain stemness properties of neuro-mesodermal axial progenitors and to sustain Fgf and Wnt signaling (van Rooijen et al., 2012; Amin et al., 2016). Finally, Cdx2 determines intestinal identity of the mid-/hindgut endoderm in embryos and allows identity maintenance of the adult gut epithelium by regulating the proliferation of stem/progenitor cells and the differentiation of mature enterocytes (Gao et al., 2009; Verzi et al., 2010; Stringer et al., 2012). Molecularly, the CDX2 protein has been shown to bind the proximal promoter of a number of target genes, as first uncovered with the intestinal sucrase-isomaltase gene (Suh et al., 1994). In addition, it also binds distant chromatin regions to prevent epigenetic silencing and keep chromatin domains open and active (Saxena et al., 2017).

While physiologically restricted to the gut epithelium in adults, CDX2 expression becomes reduced and heterogeneous in human colorectal cancer, particularly in tumors with the worst prognosis (Baba et al., 2009; Dalerba et al., 2016; Balbinot et al., 2018). This reduction facilitates tumor progression, as shown in mouse models of intestinal cancer, indicating a tumor suppressor role in the gut (Bonhomme et al., 2003; Sakamoto et al., 2017; Balbinot et al., 2018). Inversely, CDX2 is ectopically turned on outside the gut in precancerous intestine-type metaplasia and associated adenocarcinoma of foregut-derived organs including stomach and esophagus (Moskaluk et al., 2003), even though patients survival correlates with the CDX2 level in gastric cancers (Seno et al., 2002). Beside the upper digestive tract, CDX2 is also ectopically expressed in 80% of acute myeloid leukemia (AML) irrespective of the cytogenetic group but correlating with disease burden (Scholl et al., 2007). Thus, unlike the gut, CDX2 has on oncogenic effect in the hematopoietic lineage, as recently demonstrated in mice (Vu et al., 2020; Galland et al., 2021).

On this basis, the present work interrogates whether some elements of the response to CDX2 are shared during the successive steps of embryonic development in mice and subsequently whether these elements are altered in human pathologies along with CDX2.

## Results

### A Core Set of Genes Responsive To CDX2 During Mouse Development

**FIGURE 1:**
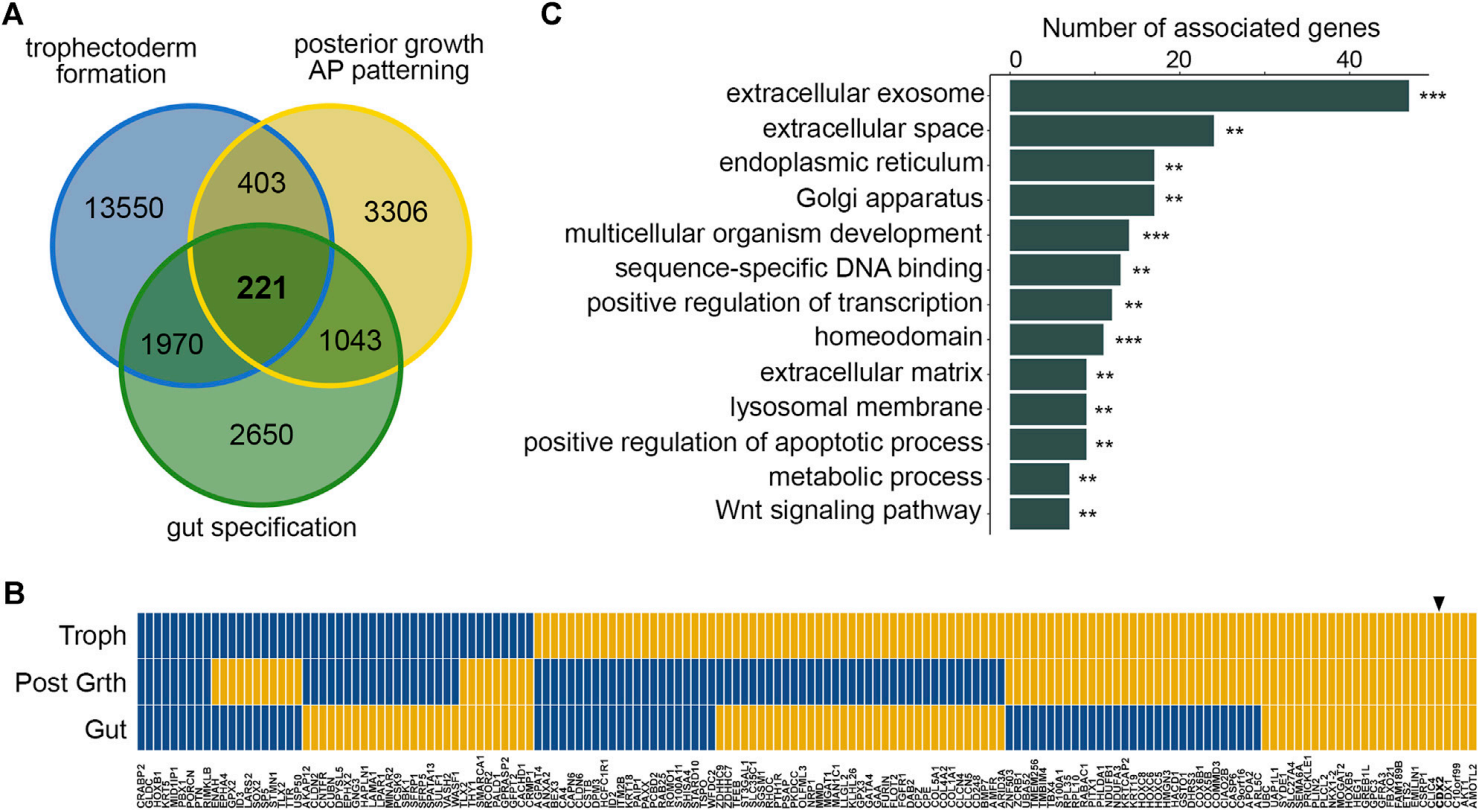
A core set of DEGs responsive to CDX2 in mouse development. (**A**) Comparison of differentially expressed genes between Cdx2-overexpressing vs wild type ES cells (trophectoderm formation; blue), E8 Cdx-null vs wild type embryos (posterior growth, AP patterning; yellow) and intestinal epithelial cells of E16 Cdx2^−/−^ vs wild type embryos (gut specification; green) showing the core set of 221 mouse DEGs. (**B**) Correlation map of the expression changes of the mouse core set of 162/221 DEGs having human orthologues, at the three developmental steps (yellow: up-regulation; blue: down-regulation): trophectoderm formation (Troph; Cdx2-overexpressing vs wild type ES cells), posterior growth (Post Grth; E8 wild-type vs Cdx-null embryos) and gut specification (Gut; E16 wild-type vs Cdx2^−/−^ embryos). At each developmental stage, the map illustrates the comparison of the Cdx2-expressing samples vs the Cdx2 non-expressing counterparts. The arrowhead shows CDX2. (**C**) Ontology analysis of the set of 162 human orthologues to the mouse core set of DEGs. **p < 0.01, ***p < 0.001.

To address if there is a common set of genes responsive to the CDX2 transcription factor during its successive functions in mouse embryogenesis, we analyzed publicly available RNAseq data related to trophectoderm formation, posterior growth, and intestinal fate determination (see Supplementary Table S1.1). For this purpose, we compared the consequences of Cdx2 overexpression in embryonic stem (ES) cells (Cambuli et al., 2014; Rhee et al., 2017), of Cdx loss of function in E8 growing embryos (Amin et al., 2016), and of Cdx2 deficiency in the intestinal endoderm of E16 embryos (Banerjee et al., 2018). With |log_2_fold-change|>2 and *p* < 0.05, a core set of 221 differentially expressed murine genes (DEGs), corresponding to 162 human orthologues, was identified in common between these three conditions (Figure 1A; Supplementary Table S1.2). Interestingly, the up or down expression changes of the DEGs were not always consistent at the three developmental steps, indicating a context-dependent response to CDX2 (Figure 1B; Supplementary Table S1.3). Ontology enrichment analysis of the 162 human orthologues revealed a significant association with “extracellular exosome”, “extracellular matrix”, “multicellular organism development”, “sequence-specific DNA binding”, “gene regulation”, “metabolic process” and “Wnt signaling” (Figure 1C; Supplementary Table S1.4). Twenty-eight genes of the DEGs core encoded nuclear proteins involved in chromatin conformation, DNA transcription and repair (Arid3a, Bmyc, Cdx1, Cdx2, Commd3, Ets2, Gata4, Hmgn3, Hoxb1, Hoxb5, Hoxc5, Hoxc6, Hoxc8, Id2, Id3, Nkx1.2, Pbx1, Prickle1, Prr13, Pitx1, Rcor2, Smarca1, Sox2, Sp5, Tbx4, Tfeb, Tlx2, Znf503), of which 11 homeobox genes known to play important roles in morphogenesis (underlined). Taken together, these results demonstrate the existence of a core set of genes responsive to CDX2 during its successive functions in embryonic development.

### A Core Set of Chromatin Sites Bound by CDX2 During Mouse Development

**FIGURE 2:**
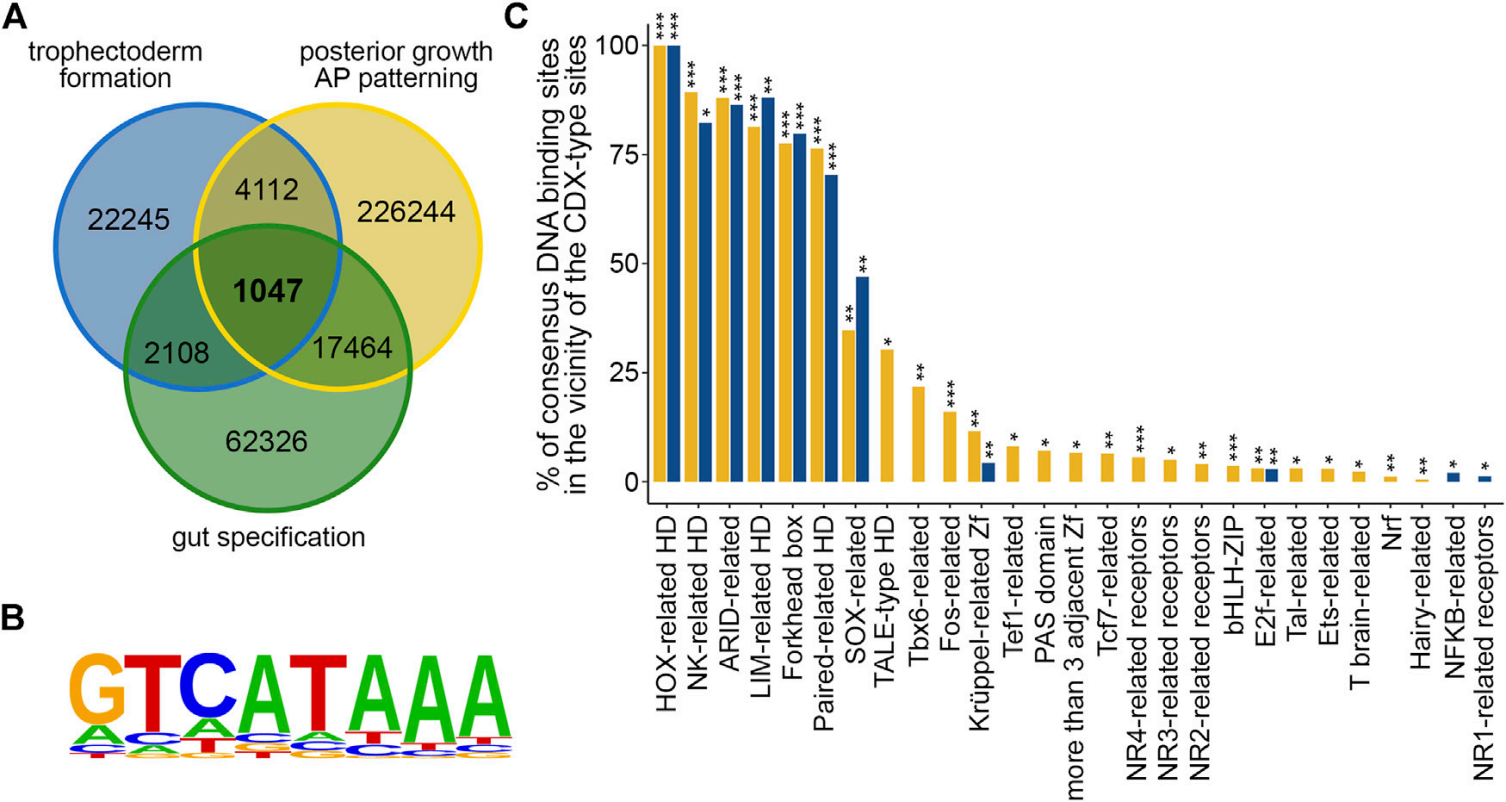
ChIPseq targets of CDX2 during mouse development. (**A**) Comparison of the CDX2-associated chromosomal regions during trophectoderm formation (trophectoderm formation; blue), embryonic posterior elongation (posterior growth, AP patterning; yellow) and gut formation (gut specification; green) showing the core of 1,047 common regions. (**B**) Consensus sequence of the 1801 CDX-type motifs present in the 1047 CDX2-bound chromosomal regions. (**C**) Enrichment in consensus binding motifs for the indicated transcription factors in the vicinity (+/50 bp) of the 1801 CDX-type sites present in the common ChIPseq regions (yellow) and in the vicinity of the 1340 CDX-type sites present in the promoters of the 221 common DEGs (blue). *p < 0.05, **p < 0.01, ***p < 0.00001. HD: homeodomain; Zf: zinc finger; h: hormone.

Next, publicly available ChIPseq data (Amin et al., 2016; Rhee et al., 2017; Banerjee et al., 2018) were used to compare the location of the CDX2 protein on chromatin at the three developmental stages analyzed above by RNAseq. It gave a core set of 1,047 chromosomal regions sharing overlapping peaks in the three conditions (Figure 2A; Supplementary Table S2.1). 265 and 466 of these peaks respectively fell into protein coding genes and their promoters (defined as the 2-kb segment upstream of the transcription start site), 52 into non-protein coding genes and their 2-kb promoters, and 264 into intergenic regions. Among the 1,047 regions, 835 (77.75%) exhibited at least one conserved motif analogous to the mouse CDX2 binding site reported in the JASPAR database (#PH0013.1), based on the functional characterization of CDX-binding sites by SELEX (T/C-A-T-A-A-A-T/G, Margalit et al., 1993). This gave a total of 1,801 CDX-type sites (enrichment *p*-value = 10^−152^) (Figure 2B; Supplementary Table S2.2). Interestingly, the ± 50 bp segments around these CDX-type sites were enriched in DNA-binding motifs for 149 transcription factors (*p* < 0.05) grouped into 25 families (Figure 2C; Supplementary Table S2.3). Moreover, 71 of these transcription factor binding motifs (*p* < 0.05), belonging to nine families, were also enriched within the ± 50 bp segments centered on the 1,314 CDX-type sites present in the promoters of the 221 DEGs (Figure 2C; Supplementary Table S2.2 and Supplementary Table S2.4). The presence of enriched binding motifs for these transcription factors nearby the CDX binding sites suggests possible direct or indirect interactions. Among the CDX2 ChIPseq peaks located in gene promoters, 8 were associated with genes of the core set of DEGs (Arid3a, Epha4, Hoxc6, Man1c1, Mgat1, Mid1ip1, Sgsm1, Tfeb), whereas 75 out of the 264 intergenic peaks (28.41%) fell into Super-Enhancer domains (Supplementary Table S2.5).

### Validation of the Core Set of Differentially Expressed Murine Genes in Independent Transgenic Mouse Models

**FIGURE 3:**
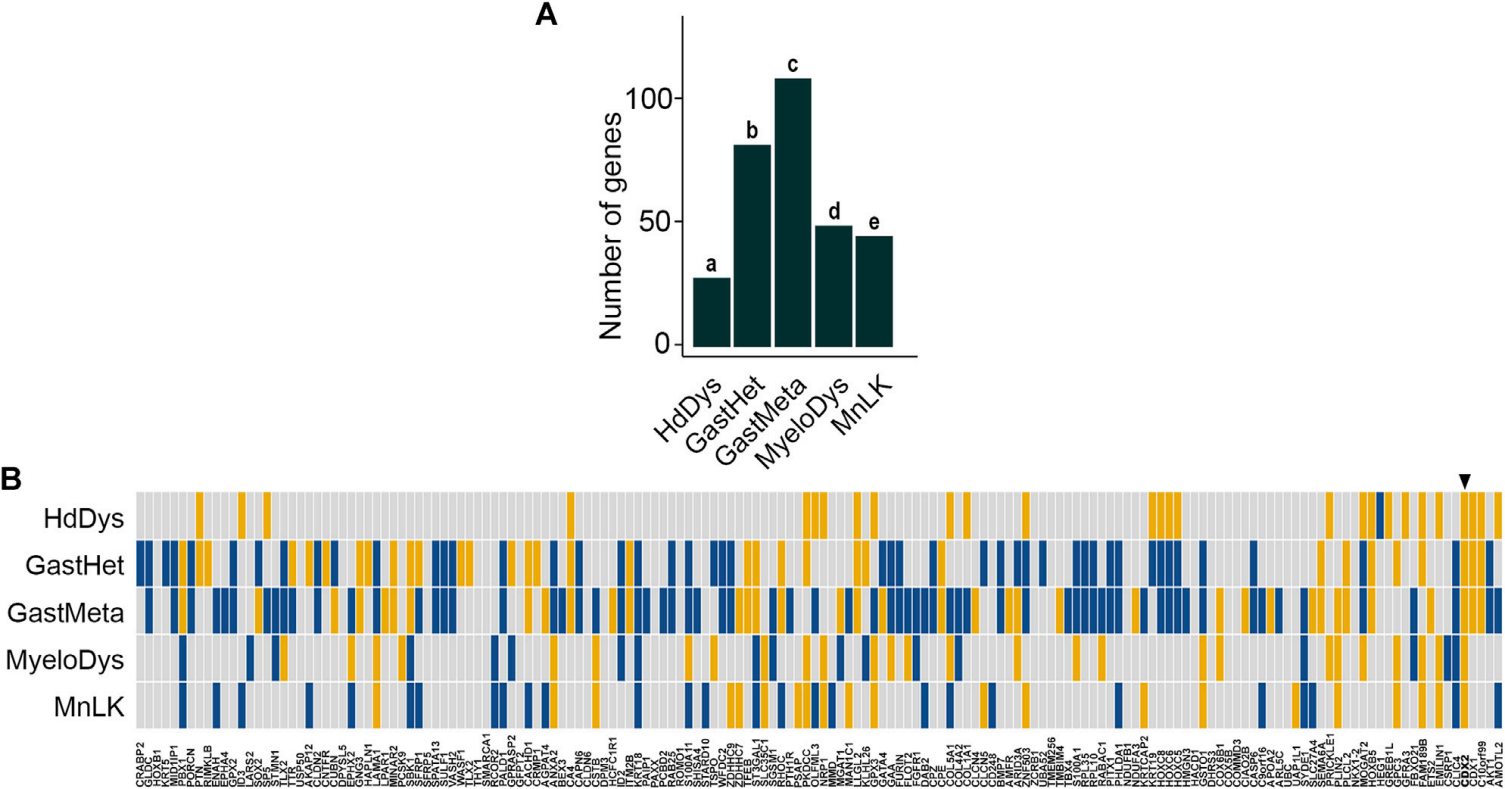
Validation of the core set of DEGs in transgenic mouse models. (**A**) Enrichment analysis of 162/221 genes of the mouse core set of DEGs having human orthologues in Cdx2-dependent transgenic mouse models of head dysgenesis (HdDys), gastric-type heteroplasia in the cecum (GastHet), gastric-type metaplasia in the pericecal region (GastMeta), myelodysplasia (MyeloDys) and monoblastic leukemia (MnLK). *p*-values are a = 5.030 E^−15^; b = 3.020 E^−12^; c = 1.578 E^−18^; d = 4.324 E^−5^; e = 1.546 E^−4^. (**B**) Correlation map of the above 162 genes of the mouse core set of DEGs in the validation models (yellow: up-regulation; blue: down-regulation). In each case, the map illustrates the comparison of the Cdx2-expressing samples vs the Cdx2 non-expressing counterparts. The arrowhead shows CDX2. The order of the genes is the same as in Figure 1B.

Five transgenic mouse models targeting the Cdx2 gene have been reported together with corresponding RNAseq data: 1) the ectopic expression of human CDX2 in the anterior epiblast at gastrulation (RsCDX2:Sox2Cre^ERT2^ embryos) resulting in severe head dysgenesis (HdDys) (Grall et al., 2019); 2) the sporadic silencing of the single wild type Cdx2 allele in heterozygous Cdx2^+/−^ embryos leading to congenital gastric-type heteroplasia in the cecum (GastHet) (Beck et al., 1999; Balbinot et al., 2018); 3) the mosaic invalidation of Cdx2 in the adult intestinal epithelium (AhCre^ERT^:Cdx2^f/f^ mice) inducing pericecal gastric-type metaplasia (GastMeta) (Balbinot et al., 2018); 4) the ectopic expression of mouse Cdx2 in hematopoietic stem cells (SclCre^ERT^:Rosa-LSL-Cdx2 mice) leading to myelodysplasia (MyeloDys) (Vu et al., 2020), and 5) the ectopic induction of human CDX2 in bone marrow stem/progenitor cells (Mx1Cre:RsCDX2 mice) inducing monoblastic leukemia (MnLK) (Galland et al., 2021). Testing the deregulated genes in these five murine models against the 162 human orthologues to the mouse core set of DEGs revealed a significant number of genes in common, namely 28 genes in HdDys (enrichment *p*-value = 5.030 E^−15^), 82 genes in GastHet (enrichment *p*-value = 3.020 E^−12^), 109 genes in GastMeta (enrichment *p*-value = 3.020 E^−12^), 45 genes in MyeloDys (enrichment *p*-value = 4.324 E^−5^) and 49 genes in MnLK (enrichment *p*-value = 1.546 E^−4^) (Figures 3A,B; Supplementary Tables S3.1–5). These results validate the core set of DEGs responsive to CDX2 in mice. In addition, they reinforce the notion of context-dependent effect.

### Pattern of the Core Set of Differentially Expressed Murine Genes in Human Pathologies

**FIGURE 4:**
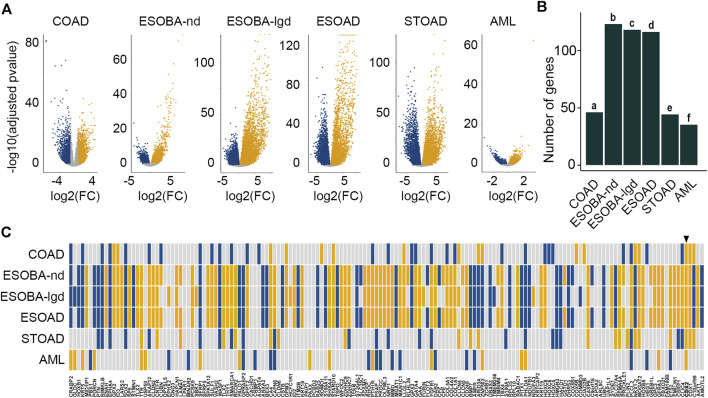
Human orthologues to the core set of mouse DEGs deregulated in diseases. (**A**) Volcano plots of the differentially expressed genes in colon adenocarcinoma (COAD), in esophageal non-dysplastic Barrett metaplasia (ESOBA-nd), low-grade dysplastic Barrett metaplasia (ESOBA-lgd) and adenocarcinoma (ESOAD), in gastric adenocarcinoma (STOAD) and in acute myeloid leukemia (AML); up-regulated (yellow), down-regulated (blue), not significant (grey). (**B**) Enrichment analysis of the set of 162 human orthologues to the mouse core set of DEGs among the differentially expressed genes in human diseases. *p*-values are a = 0.044; b = 0.16 E^−73^; c = 0.21 E^−64^; d = 0.96 E^−44^; e = 0.0028; f = 0.14 E^−4^. **(C)** Correlation map of the expression changes of the human orthologues to the mouse core set of DEGs in human diseases (yellow: up-regulation; blue: down-regulation). In each case, the map illustrates the comparison of the CDX2-high expressing samples vs CDX2 non-expressing or low-expressing counterparts. The arrowhead shows CDX2. The order of the genes is the same as in Figure 1B.

Having established and validated the core set of DEGs in mice, we addressed the pattern of the 162 orthologues in human diseases exhibiting alterations in CDX2 levels (Figure 4A; Supplementary Tables S4.1–2). Several pathological conditions were considered. First, given that the physiological expression of CDX2 is limited to the gut epithelium in adults and that it is reduced in colon cancers with bad prognosis (Balbinot et al., 2018), we compared the transcriptomes in the deciles of tumors exhibiting the lowest vs highest CDX2 levels (*n* = 44 each) among The Cancer Genome Atlas (TCGA) collection of 436 colon adenocarcinomas (COAD). Overall, a total of 46 genes among the 162 human orthologues of the core set of DEGs were differentially expressed between both groups (enrichment *p*-value = 0.044) (Figures 4B,C; Supplementary Table S4.3). Second, we considered pathological situations exhibiting abnormal ectopic expression of CDX2 outside the gut in the upper digestive tract, namely the esophagus and stomach, where ectopic CDX2 associates with precancerous metaplasia and adenocarcinoma (Moskaluk et al., 2003). In the esophagus, retrieving the list of differentially expressed genes between healthy CDX2-free mucosa (*n* = 17) and CDX2-expressing non-dysplastic Barrett metaplasia (ESOBA-nd) (*n* = 14), low-grade dysplastic Barrett metaplasia (ESOBA-lgd) (*n* = 8) and adenocarcinoma (ESOAD) (*n* = 12) (Maag et al., 2017) revealed respectively 123, 118 and 116 orthologues of the core set of DEGs (respective enrichment *p*-values are 0.16 E^−73^, 0.21 E^−64^ and 0.96 E^−44^) (Figures 4B,C; Supplementary Table S4.4–6). In the stomach, the list of differentially expressed genes in the quartiles of tumors presenting the highest vs lowest levels of CDX2 (*n* = 35 each) within the series of 272 STOAD samples of the TCGA comprised 44 DEGs of the core (enrichment *p*-value = 0.0028) (Figures 4B,C; Supplementary Table S4.7). Third, we analyzed AML in which abnormal ectopic expression of CDX2 is associated with disease burden (Scholl et al., 2007). We found 35 genes of the core set of DEGs among the genes differentially expressed between the quartiles with the highest vs lowest levels of CDX2 (*n* = 38 each) in the series of 151 AML of the TCGA (enrichment *p*-value = 0.14 E^−4^) (Figures 4B,C; Supplementary Table S4.8). Taken together, these results indicate that a significant proportion of members of the core set of CDX2-responsive genes defined during mouse development is differentially expressed in human diseases along with CDX2 changes.

## Discussion

This study identified in mice a core set of common DEGs responsive to the CDX2 homeoprotein and a core set of common chromatin sites bound to the CDX2 protein at three developmental steps at which this transcription factor plays pivotal roles: trophectoderm specification, posterior growth of the embryonic body and intestinal determination. The core of DEGs was validated in transgenic mouse models targeting Cdx2. Moreover, a significant number of human orthologues to the mouse core set of DEGs was altered in human malignancies along with CDX2. Taken together, these results show that a transcription factor, e.g., the CDX2 homeoprotein, while driving distinct functions at different steps during embryonic development, can exert a common subset of molecular activities, and that some of these activities can be subsequently deregulated in adult pathologies along with this factor.

Although studies in mice have highlighted the importance of the Cdx2 gene at many embryonic stages, developmental defects linked to alterations of this gene are rare in human, likely because its constitutive loss of function is expected to prevent trophectoderm formation and uterine implantation of the blastula. However, human CDX2 gene variants have recently been associated with sirenomelia (Lecoquierre et al., 2020), in accordance with the function attributed to this gene in posterior body elongation and patterning. Moreover, the aberrant expression of CDX2 reported in various forms of congenital endoderm-derived heteroplasia corroborates its key role in intestinal identity determination (Martin et al., 2010). Beyond embryogenesis, pathological alterations of CDX2 levels occur at its physiological site of expression, the gut, as well as ectopically in the upper digestive tract and in leukemia. The fact that the expression of a significant number of genes of the developmental core set of DEGs changed along with CDX2 in human malignancies strengthens the relevance of this DEGs core.

This study reveals that the direction of the changes of several genes of the DEGs core is not consistent at the three mouse developmental steps analyzed here, as well as in human pathologies. It emphasizes the context-dependent activity of this transcription factor. This property can be seen in view of the number of CDX2 ChIPseq peaks overlapping intergenic Super Enhancers known to control the functional activity of large chromosomal regions, and of the anti-repressing effect exerted by the CDX2 protein to prevent the incursion of inactive marks into chromatin domains and keep them accessible to other transcription partners (Verzi et al., 2013; Saxena et al., 2017). Thus, as shown in the gut, CDX2 can have inductive, permissive and repressive transcriptional effects (Verzi et al., 2013; San Roman et al., 2015; Saxena et al., 2017), indicating that its outcome depends not only on the chromatin domains that are kept open, but also on the specific repertoire of nuclear partners present in the cells and able to interact with open chromatin regions to either stimulate or inhibit transcription. Interestingly, in pathological situations the context-dependent activity of CDX2 could provide hints to explain opposite effects, being a tumor suppressor in its physiological site of expression, the gut, but an oncogene when ectopically expressed in the hematopoietic lineage. Thus, the present study opens ways to investigate novel functional interactions between developmental genes and exploit them in a therapeutic perspective.

## Materials and Methods

### Mouse and Human RNAseq and ChIPseq Data

Mouse RNAseq and ChIPseq data were retrieved from the GEO database (https://www.ncbi.nlm.nih.gov/geo/): GSE62149 (Cambuli et al., 2014) and GSE90752 (Rhee et al., 2017) for ES cells, GSE84899 (Amin et al., 2016) for E8 growing embryos, GSE115541 (Banerjee et al., 2018) for E16 intestinal endoderm, GSE123559 (Grall et al., 2019) for the head dysgenesis model, GSE89992 (Balbinot et al., 2018) for gastric-type intestinal hetero- and metaplasia, GSE133679 (Vu et al., 2020) for myelodysplasia, and GSE120487 (Galland et al., 2021) for monoblastic leukemia. The identifiers of samples used for this study are given in the Supplementary Table S1.1. Human RNAseq data from colon adenocarcinoma (COAD), stomach adenocarcinoma (STOAD) and acute myeloid leukemia (AML) were obtained from the database The Cancer Genome Atlas (The TCGA research network: https://www.cancer.gov/tcga) with the identifiers given in Supplementary Table S4.1. Esophageal metaplasia and adenocarcinoma data were from Maag et al. (2017).

### Mouse mRNAseq Read Mapping and Quantification of Expression

Quality controls of raw RNAseq reads were carried out with the FASTX toolkit (http://hannonlab.cshl.edu/fastx_toolkit/index.html) to assess base quality, nucleotide ratio and sequence duplication rate. RNAseq reads were then mapped with STAR (Dobin et al., 2013) against the mouse reference genome GRCm38. Alignments were filtered in normal mode and multi-mapped reads were discarded. For every splicing junction reconstructed from the first round of mapping, a second mapping was carried out to improve alignment. Metrics on alignment were computed with Samtools Flagstat and Samtools Stat (Danecek et al., 2021) to ensure quality of mapping. Raw gene expression, i.e. the number of mapped reads per annotated gene, were computed with HTSeq, in union mode and with the annotation of the reference genome provided as a GTF file.

### Mouse CELseq Read Mapping and Quantification of Expression

For the CELseq data of growing mouse embryos (Amin et al., 2016), sequencing adapters were trimmed with Cutadapt (Martin, 2011). Reads were mapped on the reference genome GRCm38 with BWA.aln (Li et al., 2009) and genomic coordinates were converted to alignment with BWA Samse and Samtools view. Raw read numbers were computed as described above for mRNAseq data.

### Differential Expression Analysis

For mouse ES cells data, as no replicate was available, differential expression was assessed by computing the delta of the gene expression values between control and experimental condition in each of the two datasets (Rhee et al., 2017 and Cambuli et al., 2014). Then, common differentially expressed genes between both datasets were selected with a threshold of 2 on delta. For the other mouse embryos data, namely the growing embryo (Amin et al., 2016) and the intestinal endoderm (Banerjee et al., 2018), DESeq2 (Love et al., 2014) was used. Gene expression was normalized with a regression model and differential expression was tested with the Wald test corrected by Bonferonni. False positives were identified with the Cook distance and flagged. Samples segregation was assessed by Principal Component Analysis (PCA, Supplementary Figure S1). Genes with significant variations in transcript levels were selected applying a threshold of 2 on |log2 (fold-change)| and a threshold of 0.05 on adjusted *p*-value. These genes were then compared between the datasets of the three developmental stages, i.e., ES cells, growing embryo and gut endoderm, to create the core set of common differentially expressed genes (DEGs). The enrichment in genes of the core was tested with the exact Fisher test. Orthology between the mouse DEGs and the human genome was evaluated with Ensembl Compara information based on the annotation of the mouse reference genome GRCm38, with a confidence score of 1 (high) or a minimal sequence homology of 30%, using a custom-made R-script as previously published (Mayrhofer et al., 2017). Enriched biological functions (Gene Ontology Resource, http://geneontology.org/), signaling pathways (Kyoto Encyclopedia of Genes and Genomes, https://www.genome.jp/kegg/) and protein domains (InterPro, http://www.ebi.ac.uk/interpro/) were tested on the core set of genes with DAVID (Huang et al., 2009). Further annotation of genes including symbol and description were collected with a custom-made R script.

### Mouse ChIPseq Data Processing

ChIPseq reads were mapped with BWA (Li and Durbin, 2009) against the reference genome GRCm38 as described above for the CELseq data. Unmapped reads, reads with low mapping quality, i.e., a Phred score below 30 for each base, and multi-mapped reads were filtered out. Duplicated reads were removed with GATK MarkDuplicates (Van der Auwera and O’Connor, 2020). Metrics on alignments were collected with Samtools Stats and Samtools Flagstat to ensure a good quality of read mapping (Danecek et al., 2021). Peaks were detected with MACS2 (Zhang et al., 2008). A cutoff of 10^−05^ was set on *p*-values to output peaks and significance of peaks compared to background noise was evaluated with regard to the input control. For each peak the signal was normalized computing fragment pileup per million reads. ChIPseq peaks were then selected applying a threshold of 0.05 on *p*-values and visually controlled in the genome browser IGV (Robinson et al., 2011). A core was created with ChIPseq peaks of the compared datasets overlapping with at least 10 bp in the three conditions: trophectoderm formation, antero-posterior patterning and gut specification. ChIPseq peaks were annotated with an in-house developed R script based on genes present in the annotation of the reference genome GRCm38. Both upstream and downstream genes were annotated. Intergenic ChIPseq peaks were further compared to Super-Enhancers from the database dbSUPER (Khan and Zhang, 2016).

### DNA Binding Sites Analysis

All known binding motifs of vertebrate transcription factors present in the core of ChIPseq peaks and in the gene promoters of the core of DEGs (defined as the 2-kb segment upstream of the canonical transcription start site(s) of each gene) were retrieved from the database JASPAR (Khan et al., 2018), classified with TFclass relying on “class” and “family” subdivisions (Wingender et al., 2018), and their position weight matrixes were reformatted. Enrichment for transcription factor binding motifs was tested with HOMER (Heinz et al., 2010) in the direct vicinity (+/− 50 bp) of mapped CDX-type homeobox motifs identified in the promoters of the DEGs and in the overlapping ChIPseq peaks. To test transcription factor binding motif enrichment, background sets of DNA sequences were created. These sets were composed of the same number of tested regions, i.e., promoters or overlapping ChIPseq peaks. The DNA sequences were of the same size as the tested regions and were randomly extracted from the mouse reference genome GRCm38.

### Analysis of Mouse Validation Samples and Human Pathological Samples

For samples obtained from mouse models of embryonic head dysgenesis (Grall et al., 2019), gastric-type heteroplasia and metaplasia (GastHet and GastMeta, Balbinot et al., 2018), myelodysplasia (MyeloDys, Vu et al., 2020) and monoblastic leukemia (MnLK, Galland et al., 2021), the log2 (fold-change) and *p*-value were retrieved from the literature.

For human pathological samples, raw levels of transcripts were computed with HTSeq (Anders et al., 2015) for colon adenocarcinoma (COAD), stomach adenocarcinoma (STOAD) and acute myeloid leukemia (AML). Each human gene symbol was associated to the corresponding Ensembl gene identifier and the transcript levels were normalized by computing reads per kilobase per million in order to identify groups with high and low levels of CDX2 transcripts. These groups were defined as upper and lower quartiles or deciles with a purpose of comparable size. For pair-wise comparison of groups, raw levels of transcripts were processed with DESeq2 (Love et al., 2014) as described above. Genes with significant variation in transcript levels were selected applying a threshold of 0.05 on adjusted *p*-value (Bonferroni multiple testing method). The significance of the difference in expression level of CDX2 among samples with high versus low expression of CDX2 in COAD, STOAD and AML is shown in the boxplot of the Supplementary Figure S2 and confirmed with a Wilcoxon test. For Barrett’s syndrome and esophagus adenocarcinoma, log2 (fold-change) and *p*-value were retrieved from the literature (Maag et al., 2017). For pathologies and validation datasets, the enrichment in gene of the core was tested with the one-tailed exact Fisher test with gene sets defined by significantly differentially expressed genes and a stringent gene Universe defined as genes confidently associated with a Gene Ontology.

### R-Scripts Availability

The code for the analysis of each dataset is available on github (https://github.com/victor-gourain/Gourainetal2021) and Zenodo (https://zenodo.org/badge/latestdoi/407113075).

## Data Availability

Publicly available RNAseq and ChIPseq datasets, clearly referred in the article, were used for this study.
