# Supplementary figures and images for: A Core Response to the CDX2 Homeoprotein During Development and in Pathologies
Source: Front Genet. 2021 Oct 25;12:744165. doi: 10.3389/fgene.2021.744165 (PMC8573415; doi:10.3389/fgene.2021.744165)

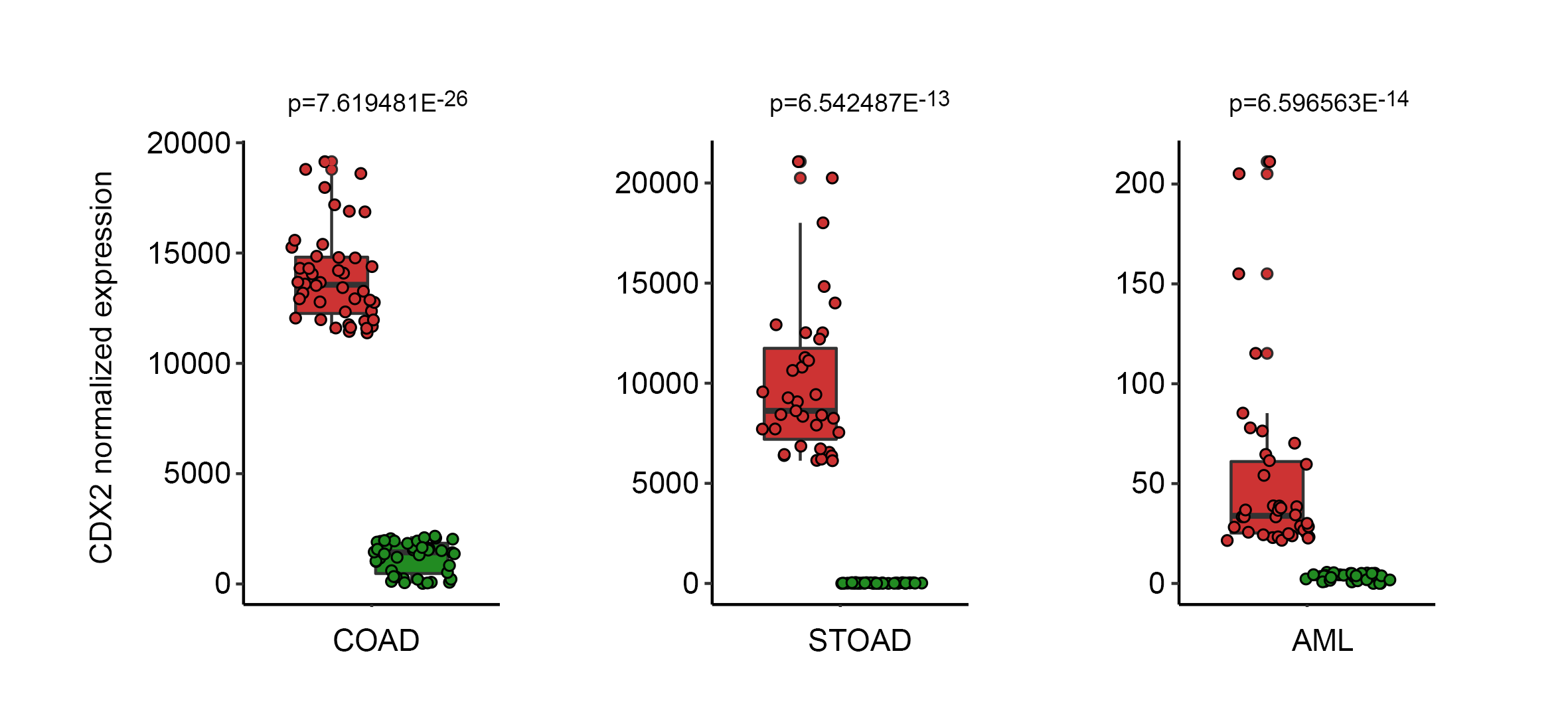

Supplement: Supplementary file 3 [file Image2.TIF]

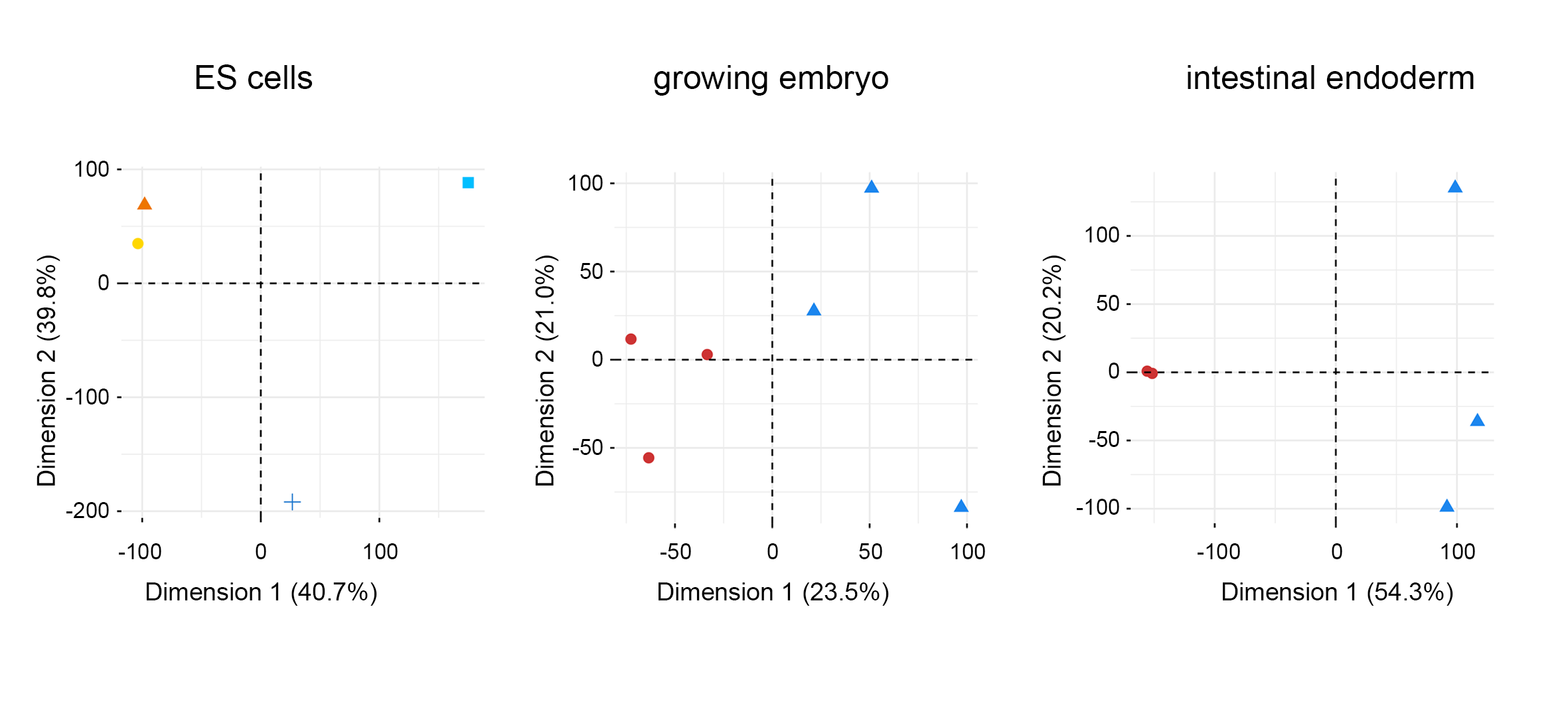

Supplement: Supplementary file 4 [file Image1.TIF]
